# Sarcoidosis with multiple organ involvement and uncommon symptoms: A case report

**DOI:** 10.1002/ccr3.6168

**Published:** 2022-08-03

**Authors:** Amirmohammad Khalaji, Somaye Sadat Rezaei, Rasoul Shajari, Maryam Masoumi

**Affiliations:** ^1^ School of Medicine Tehran University of Medical Sciences (TUMS) Tehran Iran; ^2^ Faculty of Medicine Qom University of Medical Sciences Qom Iran; ^3^ Clinical Research and Development Center Shahid Beheshti Hospital, Qom University of Medical Sciences, Shahid Beheshti Boulevard Qom Iran

**Keywords:** case report, granulomatous disease, pulmonary sarcoidosis, sarcoidosis, systemic sarcoidosis

## Abstract

Sarcoidosis is a complicated inflammatory disease characterized by the formation of non‐caseating epithelioid granulomas in many organs. Herein, we reported a sarcoidosis case with multiple organ involvements and our diagnostic criteria and treatment plan.

## Graphical abstract

A 42‐year‐old man presented with the complaint of chronic dry cough, fatigue, night sweats, dry eyes and mouth, and impotence. Herein, we report a sarcoidosis case with multiple organ involvement and uncommon presentation of impotence that was misdiagnosed with other diseases prior to presentation to our center.

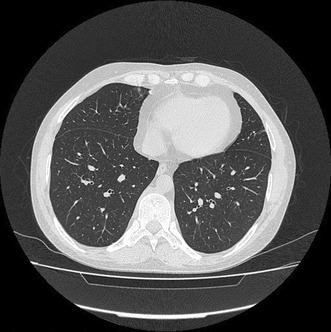

## 1 BACKGROUND

Sarcoidosis is a rare idiopathic inflammatory disease that results in the formation of granulomas in almost any body organ, which can interfere with the organs' function and structure.^1^ A joint presentation of the disease includes persistent dry cough, fatigue, and shortness of breath which can be misdiagnosed with tuberculosis, lung cancer, cat‐scratch disease, and many others.^2^ Pathologic findings of noncaseating granulomas, related clinical and radiologic findings, and exclusion of other diseases with similar findings are the three criteria for diagnosing sarcoidosis.^3^, ^4^ Asymptomatic patients often remain stable for several years and do not require treatment; but, in patients with symptomatic lungs, steroid therapy is recommended due to the overall mortality of 5% in patients who are left untreated.^2^ Due to a meta‐analysis, treatment with oral steroids (prednisolone 4–40 mg/day) leads to an improvement in chest X‐ray (CXR) over 3–24 months (Relative Risk: 1.46 [1.01 to 2.09]).^5^ This article introduces a case with sarcoidosis with chronic dry cough, weight loss, fatigue, and uncommon presentation of impotence that was misdiagnosed with other diseases prior to presentation to our center.

## 2 CASE PRESENTATION

A 42‐year‐old man with a history of mitral valve stenosis, who underwent valve replacement surgery five years ago and is currently being treated with warfarin (5 mg/day), was referred to our center with the following medical history.

**FIGURE 1:**
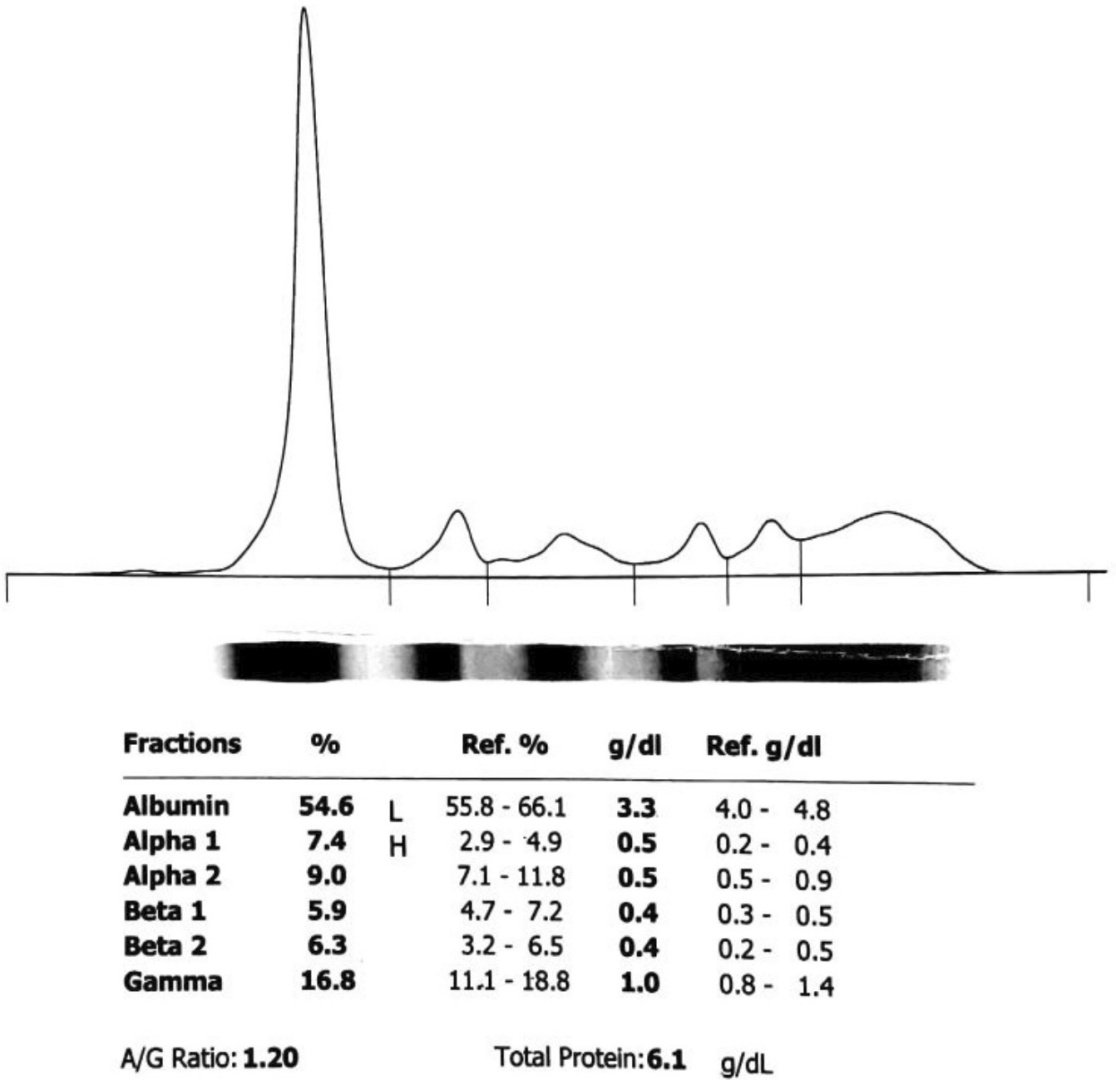
Serum protein capillary electrophoresis at first admission to the previous center; Alpha 1: Alpha1‐globulin; Alpha 2: Alpha2‐globulin; Beta 1: Beta1‐globulin; Beta 2: Beta2‐globulin; Gamma: Gamma‐globulin; A/G: Albumin/Globulin; Ref: Reference

About a year ago, the patient was referred to another center following chronic dry cough, night sweats, fatigue, and significant weight loss (approximately 10 kg). The initial laboratory assessment revealed anemia (hemoglobin [Hb] = 10.8), leukopenia (white blood cell [WBC] = 2090, neutrophil = 60%), elevated serum lactate dehydrogenase (LDH), and increased inflammatory tests, including C‐reactive protein (CRP) and erythrocyte sedimentation rate (ESR), and ferritin. Moreover, the serum ceruloplasmin level was the normal upper limit, and serum electrophoresis showed positive monoclonal IgG. Details on initial laboratory tests are reported in Table 1 and Figure 1.

**TABLE 1:** Initial laboratory at first admission to the previous center

	Concentration (%)	Reference
WBC^a^	2090 (cells/mm^3^) ↓	4500–12,500
Neutrophil	60%	
Lymphocyte	30%	
Monocyte	8%	
Eosinophil	1%	
Basophil	1%	
RBC	4.45 (× 10^6^/mm^3^)	4.4–6.1
Hemoglobin	10.8 (g/dl) ↓	14–18
Hematocrit	32.2% ↓	42–52
MCV	72.4 (fl) ↓	80–100
MCH	24.3 (pg/cell)	26–34
MCHC	33.5 (g/dl)	31–36
Platelet	182,000 (× 10^6^/dm^3^)	150,000–400,000
RDW‐CV	18.7%	11.6–14.4
Anisocytosis^b^	Positive	
Hypochromia^b^	Positive	
Microcytosis^b^	Positive	
CRP	40 (mg/L) ↑	<6
AST	92 (IU/L)	<38
ALT	85 (IU/L)	<41
ALP	1226 (U/L) ↑	80–290
LDH	750 (U/L) ↑	<480
ESR	35 (mm/h) ↑	0–15
Ceruloplasmin	61 (mg/dl)	15–60
Ferritin	433 (pg/L) ↑	24–336
Anti‐VCA IgG	Positive	
Anti‐VCA IgM	Negative	
HBsAg	Negative	
Anti‐HCV	Negative	
Blood Culture	No growth after 1, 2, and 7 days	

Abbreviations: ALP: alkaline phosphatase; ALT: alanine transaminase; AST: aspartate aminotransferase; CRP: C‐reactive protein; ESR: erythrocyte sedimentation rate; HBsAg: hepatitis B surface antigen; HCV: hepatitis C virus; LDH: lactate dehydrogenase; MCH: mean corpuscular hemoglobin; MCHC: mean corpuscular hemoglobin concentration; MCV: mean corpuscular volume; RBC: red blood cell; RDW‐CV: red blood cell distribution width; VCA: viral capsid antigen; WBC: white blood cell.

^a^ Leukopenia and WBC subtypes were confirmed by peripheral blood smear (PBS).

^b^ Anisocytosis, microcytosis, and hypochromic red blood cells were confirmed by PBS.

**FIGURE 2:**
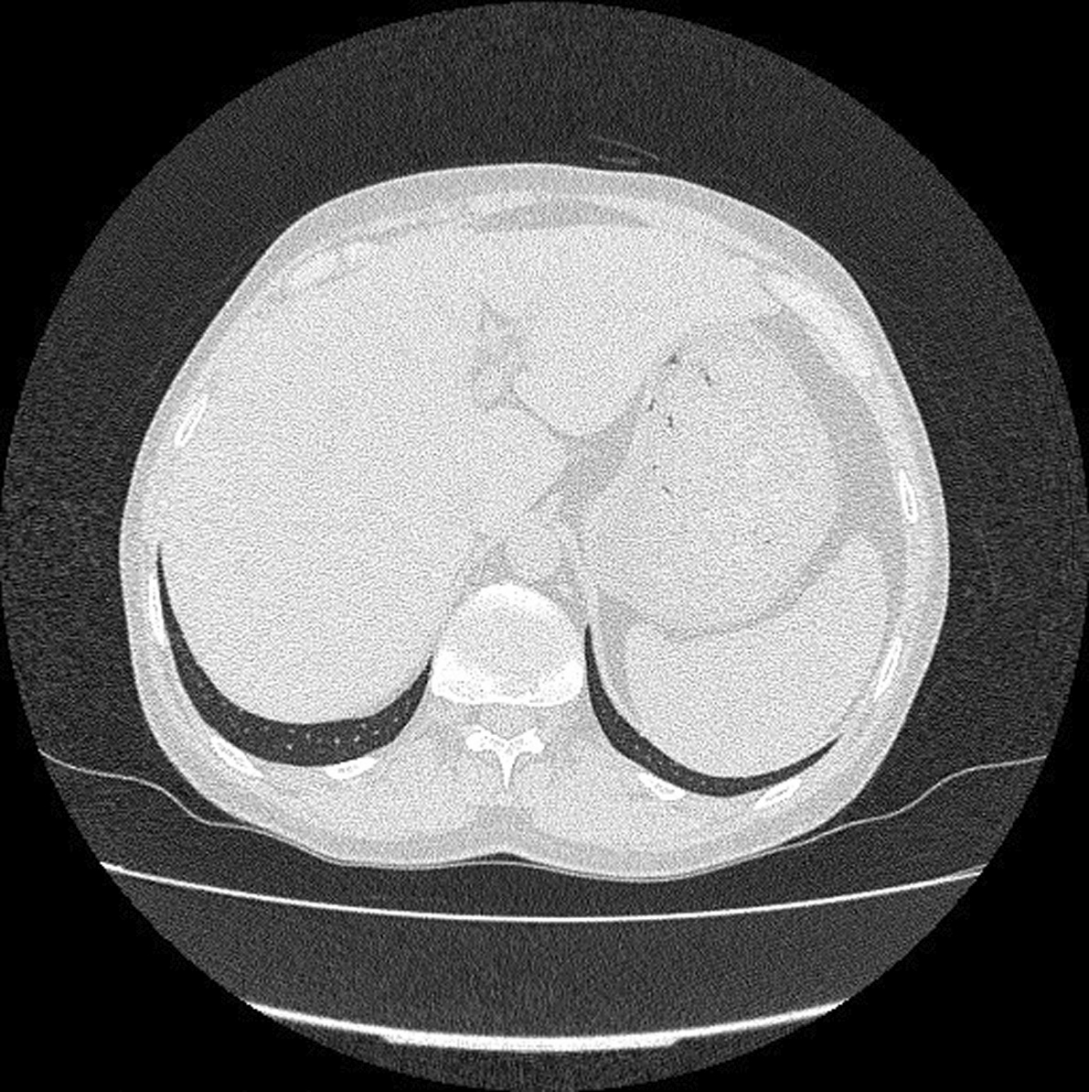
Abdominopelvic computed tomography (CT) scan confirmed hepatosplenomegaly.

During the initial workup (W/U), CXR and a subsequent spiral thoracic computed tomography (CT) scan with intravenous (IV) contrast were normal. Furthermore, according to gastrointestinal (GI) consultation, the abdominopelvic ultrasonography was performed and showed mild hepatosplenomegaly and fatty liver Grade I with no evidence of para‐aortic lymphadenopathy. A subsequent abdominopelvic CT without contrast also confirmed the sonographic findings (Figure 2). In consultation with the internal service, thyroid scintigraphy was performed and revealed subacute thyroiditis. The transesophageal echo (TEE) test was normal. The viral markers, including Hepatitis B surface antigen (Hbs Ag), anti‐hepatitis C virus antibody (HCV Ab), and anti‐human immunodeficiency virus antibody (HIV Ab), were also negative. Taking into account the results of the initial W/Us (specially bicytopenia and splenomegaly), a bone marrow biopsy and aspiration (BMB and A) was performed on the order of the hematology service to rule out malignancy. A mild megaloblastic change was shown, and the cytogenic examination was reported to be normal. Hence, the patient underwent follow‐up, and additional tests were requested.

After seven months, the patient's signs and symptoms deteriorated, and he was readmitted. The new laboratory test was performed and showed pancytopenia (WBC = 3400, Hb = 8.8, platelets [Plt] = 146,000), high titer of B_12_ (>2000), negative fluorescent antinuclear antibody (FANA), and negative wright and coomb's wright test. Furthermore, the serum gamma‐glutamyl transferase (GGT) level and the liver function tests (LFT)—aspartate aminotransferase (AST), alanine aminotransferase (ALT), and alkaline phosphatase (ALP)—were elevated significantly. Coagulation tests were also impaired. All the details are available in Table 2.

**TABLE 2:** Laboratory tests at readmission to the previous center

	Concentration (%)	Reference
WBC	3400 (cells/mm^3^) ↓	4500–12,500
Hemoglobin	8.8 (g/dl) ↓	14–18
MCV	73.9 (fl) ↓	80–100
Platelet	146,000 (× 10^6^/dm^3^)	150,000–400,000
Retic	0.9%	0.5–1.5
AST	155 (IU/L) ↑	<38
ALT	121 (IU/L) ↑	<41
ALP	5100 (U/L) ↑	80–290
LDH	577 (U/L) ↑	<480
Albumin	2.4 (g/dl)	3.5–5.5
GGT	530 (U/L) ↑	5–40
ESR	22 (mm/h) ↑	0–15
PT/INR	1.06	<1.1
BUN	10 (mg/dl)	6–24
Creatinine	0.96 (mg/dl)	0.7–1.3
Uric Acid	2.5 (mg/dl)	3.4–7
Ca	7 (mg/dl) ↓	8.6–10.3
P	2.7 (mg/dl)	2.8–4.5
Fe	39 mcg/dl↓	60–170
Ferritin	3424 ↑	24–336
TIBC	160 mcg/dl↓	240–450
Vitamin B_12_	>2000 (pg/ml) ↑	190–950
Mg	1.7 (mg/dl)	1.7–2.2
Total bilirubin	2 (mg/dl) ↑	0.1–1.2
Direct bilirubin	1.2 (mg/dl) ↑	<0.3
TIGRA (TB)	Negative	
TB‐PCR	Negative	
FANA	Negative	
Viral	Negative	
Wright and coombs wright	Negative	

Abbreviations: ALP: alkaline phosphatase; ALT: alanine transaminase; AST: aspartate aminotransferase; BUN: blood urea nitrogen; Ca: calcium; ESR: erythrocyte sedimentation rate; FANA: fluorescent antinuclear antibody; Fe: ferrous; GGT: gamma‐glutamyl transferase; LDH: lactate dehydrogenase; MCV: mean corpuscular volume; Mg: magnesium; P: phosphorus; PCR: polymerase chain reaction; PT/INR: International Normalized Ratio Prothrombin Time; TB: tuberculosis; TGRA: T‐cell interferon‐gamma release assay; TIBC: total iron binding capacity; WBC: white blood cell.

**FIGURE 3:**
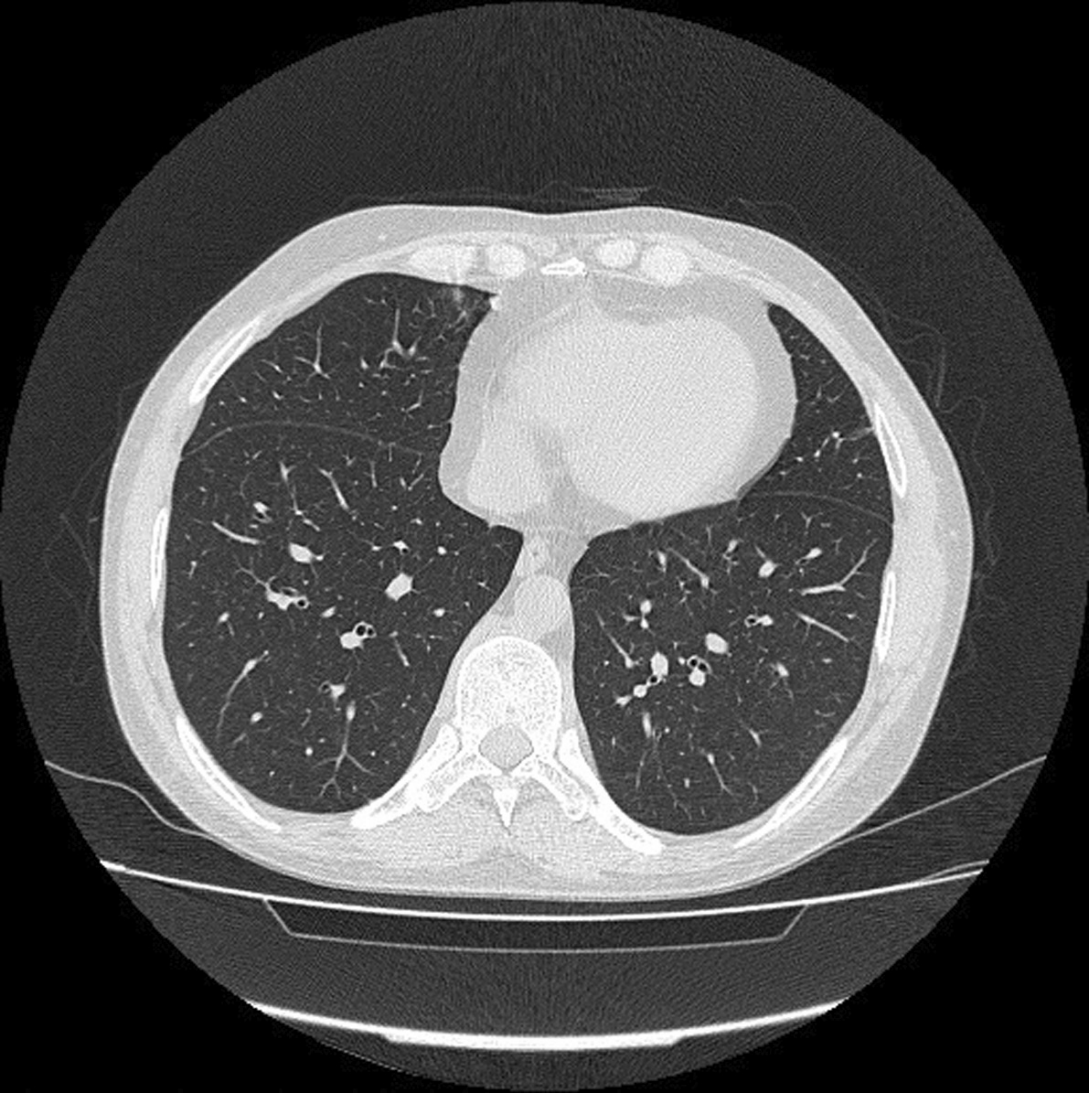
Spinal thoracic computed tomography (CT) scan without intravenous (IV) contrast. The CT scan shows mild grand glass opacity (GGO) and micronodules on the posterior side of both lungs.

Due to the persistence of constitutional symptoms, a spiral thoracic CT scan without IV contrast was taken, which showed mild grand glass opacity (GGO) in the posterior side of both lungs in addition to air trapping in the left lower lobe (Figure 3). The abdominal ultrasonography reported that echo‐free fluid was seen in the abdomen and pelvis. Moreover, tuberculosis (TB) was ruled out due to negative TB polymerase chain reaction (PCR) and interferon‐gamma release assay (IGRA). According to his abnormal tests, secondary BMB & A was performed, which showed non‐necrotizing granulomatous inflammation. The liver biopsy also showed granulomatous hepatitis besides negative Ziehl–Neelsen staining for acid‐fast microorganisms with no evidence of malignancy. As a result, the lymphoproliferative disease was ruled out, and the patient was referred to our center with the possibility of rheumatic disease.

Eventually, the patient presented to our center with the chief complaint of chronic dry cough, fatigue, night sweats, dry eyes and mouth, and impotence. His symptoms were gradually accompanied by bilateral mild (1^+^) swelling of the ankle joints and right knee, mild ascites, and bilateral slight reduced sounds in lower lung lobes during his physical examination. Of note, we did not find any skin involvement in our exams. Supplementary laboratory assessments revealed extremely high titer of angiotensin‐converting enzyme (ACE) and decreased serum C3 level beside normal level of specific immunologic tests (Details are reported in Table 3). Notably, the patient's bronchoscopy and bronchoalveolar lavage (BAL) results were normal.

**TABLE 3:** Laboratory assessments details at our center

	Concentration (%)	Reference
ACE	421 (IU/L) ↑	8–52
Lupus anti‐coagulant	Negative	
Beta‐2‐Glycoprotein (IgM)	4.3 (U/ml)	Up to 12
Beta‐2‐Glycoprotein (IgG)	<3.0 (U/ml)	Up to 10
Complement C3	0.41 (g/L) ↓	0.9–1.8
Complement C4	0.25 (g/L)	0.1–0.4
CH50	120%	70–150
FANA Titer	1:100	Up to 1:100
FANA Pattern	Nucleoli Positive	
c‐ANCA	<3.0 (AU/ml)	Up to 12
p‐ANCA	<3.0 (AU/ml)	Up to 12
Anti‐dsDNA	<10 (IU/ml)	Up to 20
Anti‐cardiolipin Antibody (IgG)	4.1 CU	Up to 10
Anti‐cardiolipin Antibody (IgM)	24.4 CU ↑	Up to 7
Anti‐SS‐A (Ro)	3.6 (U/ml)	Up to 12
Anti‐SS‐B (La)	3.2 (AU/ml)	Up to 12

Abbreviations: ACE: angiotensin‐converting enzyme; anti‐dsDNA: anti‐double stranded deoxyribonucleic acid; CH50: complement total; c‐ANCA: cytoplasmic anti‐neutrophil cytoplasmic autoantibody; FANA: fluorescent antinuclear antibody; p‐ANCA: perinuclear anti‐neutrophil cytoplasmic antibody.

Based on his outpatient follow‐ups and our findings, sarcoidosis was considered a possible diagnosis. He was treated with methylprednisolone (1 g/dose, IV) three times over 2 h, followed by prednisolone (PSL, 60 mg/day PO), CellCept (mycophenolic acid) (2 mg/day PO), calcium gluconate (1 g/day PO), and alendronate (70 mg/week PO). During the first month of follow‐up, a dramatic response to treatment was seen. His general condition was improved, so that the patient's cough and night sweats subsided, the lower extremity edema was clearly reduced, and the patient's complaint of impotence was resolved. The control laboratory tests revealed that the level of blood cells increased, whereas the level of LDH, Ferritin, ESR, CRP, and LFT decreased. Moreover, control abdominal ultrasonography reported no evidence of free abdominal fluid in favor of reducing ascites and improving the function of the patient's liver. Hence, we continued the previous treatment, tapered his PSL to 10 mg/day, and added Nolpaza (40 mg/day PO) and furosemide (40 mg/day). The patient was under a 9‐month follow‐up, and we reduced his PSL to 7.5 mg/day.

## 3 DISCUSSION

Major risk factors and primary triggers of the immune system that causes sarcoidosis is not known. Several environmental exposures such as infections, non‐infectious antigens, and metals are associated with sarcoidosis; however, the exact mechanism is still unknown.^6^ Moreover, siblings of patients with sarcoidosis showed a five times higher risk of developing sarcoidosis,^7^ which bolds the possible gene's effect on the incidence of the disease. In males, genitourinary sarcoidosis is very low and is more frequent in black men.^8^, ^9^ This report presented a case with the chief complaint of dry cough, weight loss, fatigue, night sweats, dry eyes and mouth, and impotence.

Impotence was the rarest manifestation of sarcoidosis in this case. As the erectile dysfunction (ED) in the patient was simultaneous with emerging of dry cough—the first manifestation of sarcoidosis in our case—impotence is considered as the presentation of sarcoidosis in our patient. Sarcoidosis affecting the lung is considered an interstitial lung disease (ILD). In a study, the relationship between ED and ILD was discovered, which showed that 70.4% of patients with ILD had some degree of ED.^10^ The mechanism behind ED in ILD is still unclear. However, Ibañez et al. suggested hypoxemia as a possible mechanism of ED in patients with chronic respiratory failure.^11^

Diagnosing sarcoidosis is still a challenge due to the non‐specific signs and symptoms that can be misdiagnosed easily. Sarcoidosis is a multi‐organ disease that can affect the lungs, mediastinal, hilar lymph, skin, liver, eye, and almost all organs in the body.^12^ Moreover, lung involvement is the most prevalent (about 90% of cases),^13^ mainly presenting with persistent dry cough and shortness of breath. Proper history taking, ruling out other diseases and malignancies such as lung cancer, and paraclinical tests can help clinicians diagnose sarcoidosis.

The first‐line treatment for patients with symptomatic sarcoidosis is corticosteroids.^4^ There is no evidence of a better prognosis, including less mortality, reduced long‐term symptoms, better lung function, or slower disease progression in patients who receive oral corticosteroids.^3^ However, corticosteroids are still the main treatment for patients based on expert opinion.^3^, ^4^, ^5^, ^14^ Mycophenolate mofetil (MMF, CellCept) can act as a safe and viable drug besides corticosteroids that reduce the dose of oral corticosteroid consumption, improves the clinical condition and organ functions, and radiologic findings.^15^, ^16^

The overall prognosis of sarcoidosis is good, but female gender, late‐onset, African American ethnicity, chronic phenotype, and higher stage of disease (three and four) are poor prognostic factors.^17^ In a study on Sweden's population, the mortality rate was higher in patients with sarcoidosis compared with the normal population (Hazard Ratio = 1.61), and also patients who needed treatment within the first three months had a two‐fold increase in mortality rate compared with patients who did not need any treatments.^18^ There is an increase in the trend of hospitalizations between 2007 and 2018 in the United States for respiratory failure in pulmonary sarcoidosis, but happily, inpatient mortality has decreased.^19^

## 4 CONCLUSION

Diagnosis of sarcoidosis is complicated due to the lack of specific symptoms and the variety of organs affected. Ruling out other diseases, proper history taking and physical examination, and radiologic findings can help clinicians diagnose. Corticosteroids are used as the primary treatment to alleviate symptoms and reduce progression in patients with high‐grade sarcoidosis.

## AUTHORS CONTRIBUTIONS

AK and SSR composed and revised the manuscript. RS and MM developed the research idea and revised the manuscript. All authors read and approved the final manuscript.

## CONFLICT OF INTEREST

The authors declare that they have no conflict of interest.

## CONSENT

Written informed consent was obtained from the patients for publication of this case report. A copy of the written consent is available for review by the Editor of this journal.

## Data Availability

Data sharing is not applicable to this article as no new data were created or analyzed in this study.
